# Cisplatin‐resistant cancer cells are sensitive to Aurora kinase A inhibition by alisertib

**DOI:** 10.1002/1878-0261.12066

**Published:** 2017-05-30

**Authors:** Lihong Wang, Janet Arras, Ahmed Katsha, Saif Hamdan, Abbes Belkhiri, Jeffrey Ecsedy, Wael El‐Rifai

**Affiliations:** ^1^ Department of Surgery and Cancer Biology Vanderbilt University Medical Center Nashville TN USA; ^2^ Department of Veterans Affairs Tennessee Valley Healthcare System Nashville TN USA; ^3^ Science and Engineering Department Raritan Valley Community College Branchburg NJ USA; ^4^ Translational Medicine Millennium Pharmaceuticals, Inc. Cambridge MA USA a wholly owned subsidiary of Takeda Pharmaceutical Company Limited

**Keywords:** alisertib, cisplatin, eIF4E, HDM2, MLN8237, MYC

## Abstract

*De novo* and acquired resistance to platinum therapy such as cisplatin (CDDP) is a clinical challenge in gastric cancer treatment. Aberrant expression and activation of aurora kinase A (AURKA) and eukaryotic translation initiation factor 4E (eIF4E) are detected in several cancer types. Herein, we investigated the role of AURKA in CDDP resistance in gastric cancer. Western blot analysis demonstrated overexpression of AURKA and phosphorylation of eIF4E in acquired and *de novo* CDDP‐resistant gastric cancer models. Inhibition of AURKA with MLN8237 (alisertib) alone or in combination with CDDP significantly suppressed viability of CDDP‐resistant cancer cells (*P* < 0.01). Additionally, inhibition or knockdown of AURKA decreased protein expression of p‐eIF4E (S209), HDM2, and c‐MYC in CDDP‐resistant cell models. This was associated with a significant decrease in cap‐dependent translation levels (*P *< 0.01). *In vivo* tumor xenografts data corroborated these results and confirmed that inhibition of AURKA was sufficient to overcome CDDP resistance in gastric cancer. Our data demonstrate that AURKA promotes acquired and *de novo* resistance to CDDP through regulation of p‐eIF4E (S209), c‐MYC, HDM2, and cap‐dependent translation. Targeting AURKA could be an effective therapeutic approach to overcome CDDP resistance in refractory gastric cancer and possibly other cancer types.

**Abbreviations**
AURKA: aurora kinase A
CDDP: cisplatin or cis‐diamminedichloroplatinum
CDDPR: cisplatin resistant
eIF4E: the eukaryotic translation initiation factor 4E
HDM2: human double minute 2
MLN8237: alisertib
siAURKA: small‐interfering RNA of AURKA
siControl: small‐interfering RNA of scramble
sieIF4E: small‐interfering RNA of eIF4E

## 1 Introduction

Gastric cancer is currently the fourth most common malignancy worldwide and the third leading cause of cancer‐related deaths in both males and females (Ferlay *et al*., 2015). Resistance to chemotherapeutic agents is a major clinical problem in the management of patients with cancer. In fact, patients with gastric cancer exhibit poor response to neoadjuvant and adjuvant chemotherapy due to intrinsic and acquired drug resistance (Hohenberger and Gretschel, 2003; Jemal *et al*., 2011). Platinum‐based regimens that include cisplatin [cis‐diamminedichloroplatinum (CDDP)] are employed to treat a variety of cancers such as ovarian, bladder, esophageal, and gastric cancers. Although platinum‐based chemotherapeutic regimens have become standard approaches for the treatment of patients with advanced gastric cancer, there is a relatively low rate of complete response (Van Cutsem *et al*., 2008). Therefore, there is an urgent need for the development of novel therapeutic approaches based on understanding the underlying molecular pathways mediating cisplatin resistance.

Aurora kinase A (AURKA) is a serine/threonine cell cycle kinase, whose main physiological function in normal cells is related to centrosome and spindle assembly (Lens *et al*., 2010). We and others have reported amplification and overexpression of AURKA in several malignancies including gastric, esophageal, colon, breast, and ovarian cancers (Aradottir *et al*., 2015; Cirak *et al*., 2015; Dar *et al*., 2008b, 2009; Fang *et al*., 2011; Furukawa *et al*., 2006; Gritsko *et al*., 2003; Katayama *et al*., 1999; Katsha *et al*., 2013, 2015; Zheng *et al*., 2016). Recent studies have shown that inhibition of AURKA can sensitize cancer cells to CDDP in gastric cancer (Sehdev *et al*., 2012) and its overexpression may predict platinum resistance in epithelial ovarian cancer (Mignogna *et al*., 2016). It has been shown that AURKA can inhibit p53 through direct binding and activation of the E3‐ubiquitin ligase, human double minute 2 (HDM2) (Sehdev *et al*., 2014). Notably, AURKA overexpression in cancer cells has also been implicated in activating a plethora of prosurvival oncogenic pathways including β‐catenin, NF‐κB, and AKT (Dar *et al*., 2008b, 2009; Katsha *et al*., 2013). These studies suggest that AURKA overexpression in cancer cells has prosurvival and antiapoptotic functions.

The eukaryotic translation initiation factor 4E (eIF4E), which is the rate‐limiting component in formation of eIF4F complex (Jones *et al*., 1997), is involved in recognizing the 5′ 7‐methyl guanosine cap of mRNAs (Mamane *et al*., 2004). Regulation of protein translation is important for the control of cell growth and proliferation. Aberrant activation of eIF4E has been linked to resistance to therapy and poor outcome in breast, melanoma, prostate, and gastric cancers (Chen *et al*., 2004; Graff *et al*., 2009; Pettersson *et al*., 2015; Zhan *et al*., 2015). Activity of eIF4E is dependent upon its phosphorylation on Ser209, leading to oncogenic transformation through translational control (Wendel *et al*., 2007). Enhanced translation of growth‐promoting genes such as c‐MYC, cyclin D1, and VEGF has been associated with the activation of eIF4E. Activation of eIF4E has been implicated in chemotherapeutic resistance in several cancer types (Kraljacic *et al*., 2011; Martinez‐Marignac *et al*., 2013; Zhan *et al*., 2015). A recent study reported that inhibiting eIF4E might be a viable therapeutic approach to overcome resistance to vemurafenib, BRAF inhibitor, in melanoma (Zhan *et al*., 2015). Therefore, targeting eIF4E is considered a promising anticancer strategy to combat drug resistance (reviewed in Siddiqui and Sonenberg, 2015).

In this study, we demonstrate that AURKA promotes acquired and *de novo* resistance to cisplatin in *in vitro* and *in vivo* gastric cancer cell models. We show that AURKA mediates phosphorylation of eIF4E to promote protein translation of pro‐oncogenic downstream effectors such as c‐MYC and HDM2. We propose targeting AURKA as an effective second‐line therapeutic approach in cisplatin‐resistant cancers.

## 2 Materials and methods

### 2.1 Cell culture and reagents

Human gastric adenocarcinoma cell lines (AGS, SNU‐1, MKN28, and MKN45) were maintained in Dulbecco's modified Eagle's medium (GIBCO, Carlsbad, CA, USA). All cell lines were authenticated using short tandem repeat (STR) profiling (Genetica DNA Laboratories, Burlington, NC, USA). The cell lines were supplemented with 10% fetal bovine serum (Invitrogen Life Technologies, Carlsbad, CA, USA) and with 1% penicillin/streptomycin (GIBCO). The investigational AURKA inhibitor alisertib, known as MLN8237 (Millennium Pharmaceuticals, Inc., Cambridge, MA, USA), was used for *in vitro* and *in vivo* studies. The AURKA expression plasmid was generated as described previously (Dar *et al*., 2009). Specific antibodies against p‐AURKA (T288), AURKA, p‐eIF4E (S209), eIF4E, and β‐actin were purchased from Cell Signaling Technology (Beverly, MA, USA). Specific antibody against c‐MYC was purchased from Santa Cruz Biotechnology (Dallas, TX, USA). Transfection reagent LipoJet was purchased from SignaGen (Gaithersburg, MD, USA). Cisplatin (CDDP) (APP Pharmaceuticals, LLC) was obtained from Vanderbilt University Medical Center pharmacy in a stock solution (2 mmol·L^−1^), prepared using 1 × PBS.

### 2.2 Establishment of CDDP‐resistant sublines from AGS cells

AGS CDDP‐resistant cells were established by continuous exposure to CDDP starting at 0.1 μm and increasing in a stepwise manner to 10 μm for 6 months. Finally, we generated two AGS CDDP‐resistant pools (Pools 1 and 2) from which we selected two highly resistant single clones (Clones 1 and 2) to be used in the study.

### 2.3 Western blotting

Cells were scraped on ice and centrifuged, and pellets were resuspended in RIPA lysis buffer. Cell lysates were placed on ice. Protein concentration was determined using Bradford Protein Assay (Bio‐Rad Laboratories, Hercules, CA, USA). Proteins (25 μg) from each sample were subjected to SDS/PAGE and transferred onto nitrocellulose membranes using the semidry transfer protocol (Bio‐Rad Laboratories). After transfer, membranes were probed with each respective primary antibody overnight at 4 °C. Following incubation, the membranes were probed with HRP‐conjugated secondary antibodies (Cell Signaling). Protein bands were visualized using the commercial Immobilon Western Chemiluminescent HRP Substrate Kit (Millipore, Billerica, MA, USA).

### 2.4 AURKA and eIF4E silencing by small‐interfering RNA (siRNA)

Cells were seeded at 60% confluency in 10% FBS‐containing DMEM for 24 h in p60 plates. AURKA and eIF4E were transiently silenced by using siAURKA and sieIF4E (Invitrogen) for a total of 48 h. A negative siRNA control (Ambion, Austin, TX, USA) was used in each experiment. Transfection of cells was achieved by using a LipoJet reagent (SignaGen) according to the manufacturer's instructions. Following 24‐h transfection, medium was replaced with DMEM, supplemented with 5% FBS and antibiotics for another 24 h prior to harvesting. Validation of AURKA and eIF4E knockdown was assessed by qPCR and western blot analyses.

### 2.5 AURKA overexpression

The expression plasmid for AURKA was generated as described previously (Dar *et al*., 2009). A synthetic Flag‐tag sequence was added at the N terminus of AURKA. The recombinant adenovirus expressing AURKA or control was generated as described previously (Katsha *et al*., 2013). The recombinant adenovirus was generated by cotransfecting human embryonic kidney (HEK)‐293 cells (American Tissue Culture Collection) with the shuttle and adenoviral backbone (pJM17) plasmids using the Calcium Phosphate Transfection Kit (Applied Biological Materials, Inc., Richmond, BC, USA).

### 2.6 CellTiter‐Glo Luminescence Assay

CellTiter‐Glo assay was used to determine IC50 and drug dose–response curves for each cell line following treatment with CDDP, alisertib (MLN8237), or in combination. Cells were seeded at 2000 cells/well in a 96‐well plate. Cells were treated with CDDP with or without MLN8237 following a 12 × 2‐fold serial dilution treatment in 5% FBS‐containing DMEM. After five days, cell viability was measured using CellTiter‐Glo Luminescence Assay (Promega, Madison, WI, USA). The dose–response curves were fitted using GraphPad Prism 5, following a nonlinear regression (four‐parameter, least‐squares fit) method. IC50 values were determined by a four‐parameter, nonlinear regression method. The data were generated from at least three independent experiments.

### 2.7 RNA extraction and real‐time RT‐PCR

Cells were scraped and centrifuged, and total RNA was isolated using the RNeasy Mini kit (Qiagen, Germantown, MD, USA), and cDNA synthesis was performed using an iScript cDNA Synthesis Kit (Bio‐Rad). Specific primers’ sequences were acquired from qPrimerDepot‐A quantitative real‐time PCR primer database (https://primerdepot.nci.nih.gov/). All primers were purchased from Integrated DNA Technologies (IDT), Coralville, IA, USA). c‐MYC‐F: CACCGAGTCGTAGTCGAGGT; c‐MYC‐R: TTTCGGGTAGTGGAAAACCA; AURKA‐F: AGTTGGAGGTCCAAAACGTG; AURKA‐R: TCCAAGTGGTGC ATATTCCA; HDM2‐F: ACCTCACAGATTCCAGCTTCG; HDM2‐R: TTTCATAGTATAAGTGTCTTTTT. The quantitative RT‐PCR was performed using a Bio‐Rad CFX Connect Real‐time System, with the threshold cycle number determined by Bio‐Rad cfx manager software version 3.0 (Bio‐Rad Life Sciences, Hercules, CA, USA). Reactions were performed in triplicate, and the threshold cycle numbers were averaged. The results of the genes were normalized to HPRT1 housekeeping gene.

### 2.8 Luciferase reporter assay

For the cap‐dependent dual luciferase reporter assays, cells were transfected with 1 μg of a dual‐*renilla*‐firefly‐luciferase pcDNA3‐rLuc‐PolioIRES‐fLuc reporter (a kind gift from John Blenis, Harvard Medical School), to measure cap‐dependent/cap‐independent translation (Dos Santos *et al*., 2016). Cells were plated in six‐well plates, transfected using Lipofectamine 2000 reagent (Life Technologies), and treated with MLN8237 for 24 h. The lysates were prepared in triplicate, and the dual luciferase reporter assay (Promega) was used following the manufacturer's protocol. The rate of cap‐dependent translation was defined as the ratio of renilla to firefly luciferase activities.

For c‐MYC transcription activity, cells were cotransfected with 250 ng of β‐GAL and 1 μg of 4xEMS c‐MYC reporter (a kind gift from Stephen R. Hann, Vanderbilt University School of Medicine) in six‐well plates using the PolyJet transfection reagent (SignaGen). As a positive control, parental cells were transfected with 500 ng of pcDNA‐c‐MYC plasmid in addition to 250 ng of β‐GAL and 1 μg of 4xEMS c‐MYC reporter. Cells were treated with MLN8237 for 24 h and then lysed to measure luciferase activity using the Luciferase Assay Kit (Promega). The c‐MYC luciferase activities were normalized to β‐GAL levels.

### 2.9 *In vivo* tumor xenograft

All animal work was approved by the Vanderbilt Institutional Animal Care and Use Committee. MKN45 cells (2 × 10^6^) suspended in 200 μL of DMEM and Matrigel mixture (50% DMEM supplemented with 10% FBS and 50% Matrigel) were injected into the flank regions of female 201 NIH III HO nude mice (Charles River Laboratories, Wilmington, MA, USA). We used eight mice per group. The tumors were allowed to grow until 150–200 mm^3^ in size before starting treatment with CDDP (2.5 mg·kg^−1^ body weight, once a week, IP) alone, MLN8237 (40 mg·kg^−1^, five times per week, orally) alone, or the combination of CDDP and MLN8237 for 28 days. Tumor xenografts were measured every three days, and tumor size was calculated according to the following formula: T vol = *L* × *W* ^2^ × 0.5, where T vol is tumor volume, *L* is tumor length, and *W* is tumor width. For control group, mice were sacrificed when tumor size reaches 1000 mm^3^ in accordance with the approved protocols. At the end of treatment, three to six xenograft tumors from each group were collected and processed for western blot (p‐AURKA (T288), AURKA, p‐eIF4E (S209), eIF4E, c‐MYC). Immunohistochemical analysis was carried out on formalin‐fixed, paraffin‐embedded tissues to measure Ki‐67 and cleaved caspase 3 protein expression levels. Ki‐67 and cleaved caspase 3 protein expression levels were evaluated by imagej software (NIH, Bethesda, MD, USA). Relative integrated density indicates the quantification data of diaminobenzidine staining signal analyzed by ImageJ IHC Toolbox plugin (https://imagej.nih.gov/ij/plugins/ihc-toolbox/index.html; Zhang *et al*., 2016).

### 2.10 Statistical analysis

Data are presented as means ± standard error of mean. Statistical significance of difference between control groups and treatment groups was determined using one‐way ANOVA test. Statistical analyses were carried out using graphpad prism 5 software, nonlinear regression (GraphPad Software Inc., La Jolla CA, USA). The correlation between two parameters was determined by two‐tailed Student's test. The differences were considered statistically significant when the *P* < 0.05.

## 3 Results

### 3.1 Acquired resistance to CDDP correlates with high levels of AURKA and p‐eIF4E proteins in gastric cancer cells

**Figure 1:**
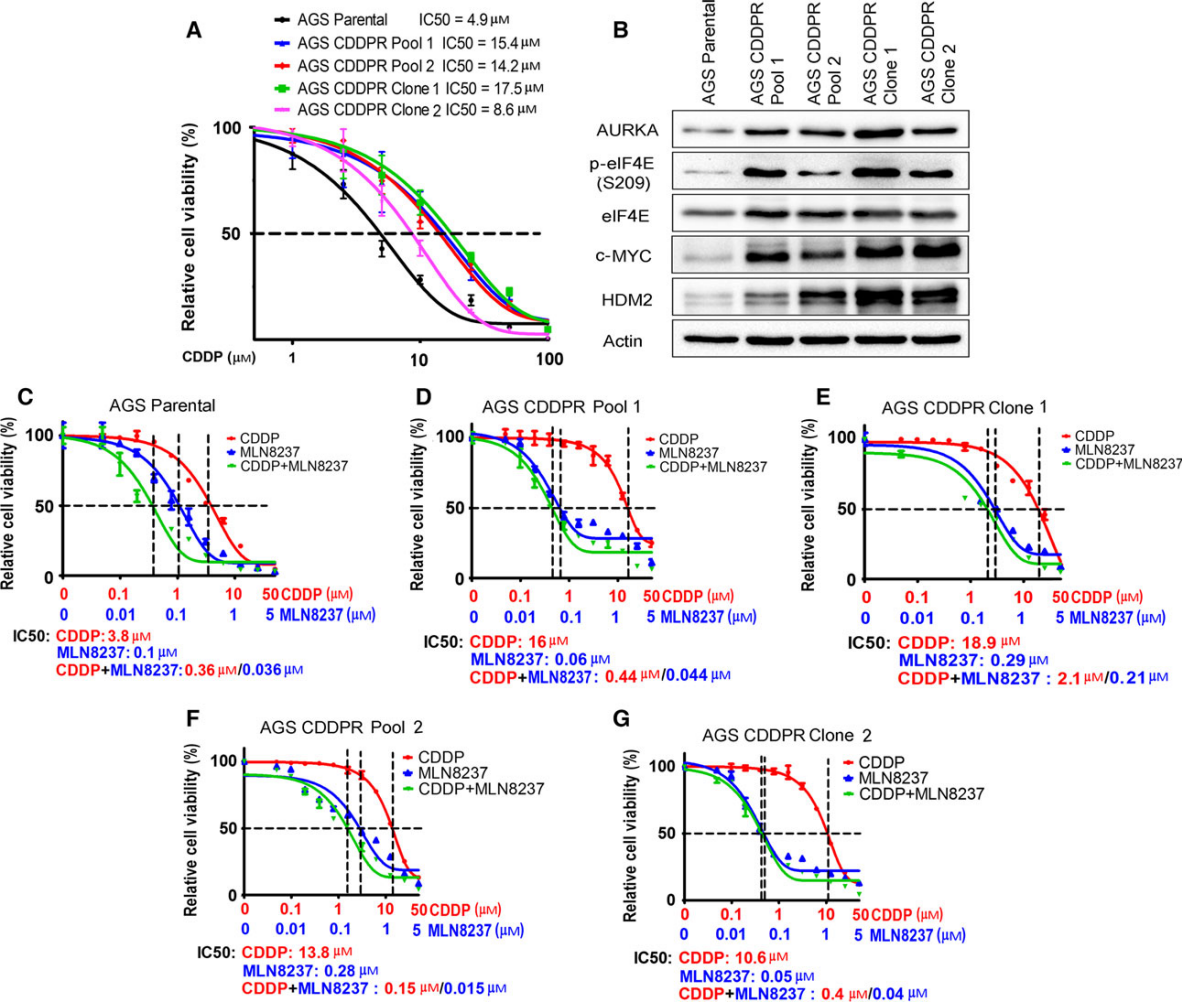
CDDP‐resistant cells express high levels of AURKA and p‐eIF4E (S209) proteins. (A) AGS Parental, AGS CDDP‐resistant (CDDPR) Pool 1, AGS CDDPR Pool 2, AGS CDDPR Clone 1, and AGS CDDPR Clone 2 cells in 96‐well plates were treated with CDDP following a 8‐point twofold serial dilution, starting at 100 μm concentration. Cell viability was measured using CellTiter‐Glo assay. Dose response was fitted using a three‐parameter nonlinear regression method. (B) Cell lysates from AGS Parental and CDDP‐resistant cell lines were subjected to western blot analysis of the indicated proteins. Levels of AURKA, p‐eIF4E (S209), c‐MYC, and HDM2 proteins were substantially higher in AGS CDDP‐resistant cells than in AGS Parental cells. AGS Parental (C), AGS CDDPR Pool 1 (D), AGS CDDPR Clone 1 (E), AGS CDDPR Pool 2 (F), and AGS CDDPR Clone 2 (G) cells in 96‐well plates were treated with MLN8237 alone, CDDP alone, or CDDP + MLN8237 at a fixed ratio (10 : 1) following a 12‐point twofold serial dilution. Cell viability was measured and dose–response curves were generated as in (A). The mean value of IC50 of each drug treatment was generated from at least three independent experiments.

Cisplatin‐based chemotherapeutic regimen is a standard approach for the treatment of patients with gastric cancer. Unfortunately, disease progression eventually occurs because of acquired drug resistance (reviewed by Lu *et al*., 2016). To determine the molecular mechanism that drives acquired resistance to CDDP in gastric cancer cells, we generated AGS cell models of acquired resistance to CDDP using a stepwise increase in CDDP concentrations starting at 0.1–10 μm for 6 months. At the end of CDDP treatments, we obtained the following CDDP‐resistant AGS cell models: AGS CDDPR Pool 1, AGS CDDPR Pool 2, AGS CDDP Clone 1, and AGS CDDPR Clone 2. Short‐term cell viability (5 days) in response to CDDP treatments was evaluated by CellTiter‐Glo assay. The CDDP IC50s of AGS CDDPR Pool 1 (15.4 μm), AGS CDDPR Pool 2 (14.2 μm), AGS CDDPR Clone 1 (17.5 μm), and AGS CDDPR Clone 2 (8.6 μm) were significantly higher than that of AGS Parental cells (4.9 μm,* P* < 0.05) (Fig. 1A), confirming that AGS CDDP‐resistant cell models conferred the CDDP resistance phenotype. Using western blot analysis, we found that CDDP‐resistant AGS cells gained overexpression of AURKA, p‐eIF4E (S209), c‐MYC, and HDM2 proteins, as compared to AGS Parental cells (Fig. 1B).

### 3.2 Cancer cells with acquired resistance to CDDP are sensitive to AURKA inhibition

To find out whether targeting AURKA can reverse these signaling effects and restore therapeutic efficacy in CDDP‐resistant cells, we inhibited AURKA with MLN8237, an investigational AURKA‐specific inhibitor, alone or in combination with CDDP in AGS Parental and CDDPR cells. Using short‐term cell viability (5 days) CellTiter‐Glo assay, the results showed that MLN8237 alone significantly decreased cell survival (*P* < 0.001) in AGS Parental cells, AGS CDDPR Pool 1, AGS CDDPR Pool 2, AGS CDDPR Clone 1, and AGS CDDPR Clone 2 cells in a dose‐dependent manner. Interestingly, while the combination of MLN8237 and CDDP had an additive effect on parental cells (Fig. 1C), the addition of CDDP to MLN8237 had minimal advantage over MLN8237 alone in CDDP‐resistant cells (Fig. 1D–G). Collectively, the data indicated that inhibition of AURKA using MLN8237 is sufficient to decrease cell viability of AGS CDDP‐resistant cells.

### 3.3 AURKA promotes CDDP‐acquired resistance through regulation of eIF4E, c‐MYC, and HDM2

**Figure 2:**
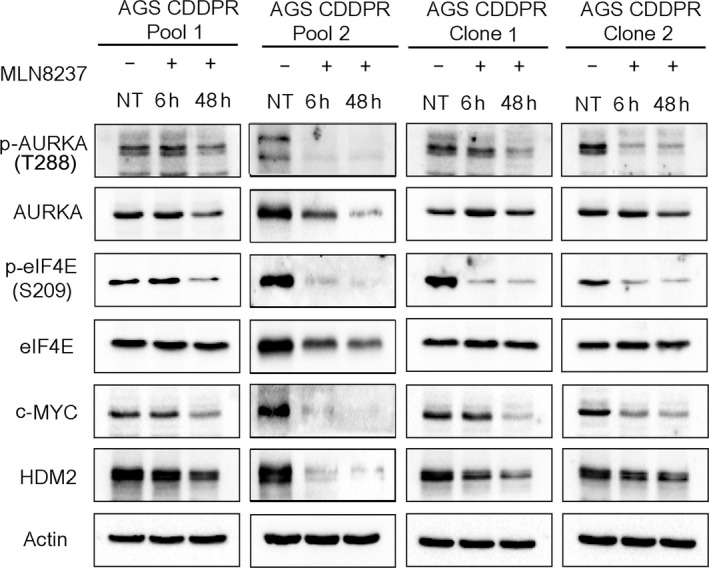
Pharmacologic inhibition of AURKA with MLN8237 downregulates phosphorylation of eIF4E and protein expression of downstream effectors in CDDP‐resistant cells. AGS CDDPR Pool 1, AGS CDDPR Pool 2, AGS CDDPR Clone 1, and AGS CDDPR Clone 2 cells were treated with MLN8237 (0.5 μm), and cell lysates were subjected to western blot analysis of the indicated proteins. The data showed that pharmacologic inhibition of AURKA with MLN8237, as indicated by decreased protein levels of p‐AURKA (T288), reduced the protein levels of p‐eIF4E (S209) and its protein translation targets, c‐MYC and HDM2 in CDDP‐resistant cells. Gel loading was normalized for equal β‐actin.

**Figure 3:**
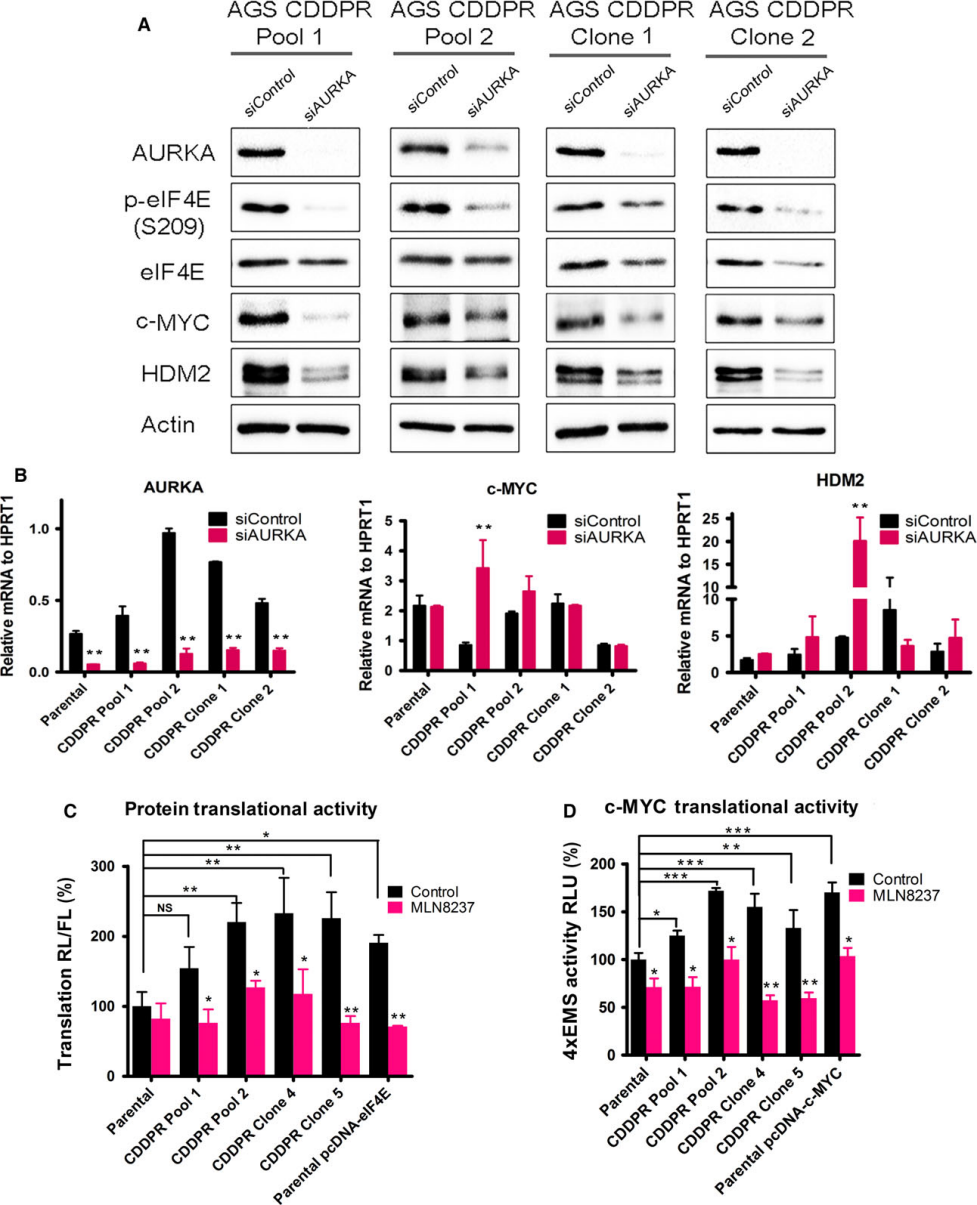
Genetic knockdown of AURKA reduces phosphorylation of eIF4E and protein levels of its downstream effectors in CDDP‐resistant cells. AGS CDDPR Pool 1, AGS CDDPR Pool 2, AGS CDDPR Clone 1, and AGS CDDPR Clone 2 cells were transfected with control siRNA (siControl) or siRNA specific for AURKA (siAURKA) for 48 h. Cell lysates and total RNA were subjected to western blot (A) and real‐time RT‐PCR (B) analyses, respectively. The data showed that knockdown of AURKA led to decreased p‐eIF4E (S209), c‐MYC, and HDM2 protein levels, but did not decrease mRNA expression of the corresponding encoding genes in CDDP‐resistant cells. Gel loading was normalized for equal β‐actin. HPRT1 housekeeping gene was used as the internal control for PCR. (C) AGS Parental, AGS CDDPR Pool 1, AGS CDDPR Pool 2, AGS CDDPR Clone 1, and AGS CDDPR Clone 2 cells were transfected with luciferase reporter plasmid (pcDNA3‐rLuc‐Polio IRES‐fLuc). Cells were treated with MLN8237 (0.5 μm) for 24 h. The rate of cap‐dependent translation was defined as the ratio of renilla to firefly luciferase activities. (D) AGS Parental, AGS CDDPR Pool 1, AGS CDDPR Pool 2, AGS CDDPR Clone 1, and AGS CDDPR Clone 2 cells were transfected with 4 × EMS c‐MYC reporter. Cells were treated with MLN8237 (0.5 μm) for 24 h. Luciferase activity was determined as described in Materials and methods. The results showed that the basal level of cap‐dependent translation activity and c‐MYC transcription activity in AGS CDDPR cells are significantly higher than in AGS Parental cells. ***P <* *0.001. Additionally, treatment with MLN8237 significantly reduced cell cap‐dependent translation or 4xEMS c‐MYC Luciferase activity in comparison with the no‐treatment control group. Transient transfections with pcDNA‐eIF4E (C) or pcDNA‐c‐MYC (D) were included as controls. *P <* *0.05, **P <* *0.01, ***P <* *0.001.

Based on our results indicating that AURKA is important for CDDP‐acquired resistance (Fig. 1), and the positive correlation between the protein expression of AURKA, p‐eIF4E (S209), c‐MYC, and HDM2 in CDDP‐resistant cells (Fig. 1B), we examined whether AURKA inhibition by MLN8237 can regulate these signaling molecules. Indeed, we observed a substantial reduction in the protein levels of p‐eIF4E (S209), c‐MYC, and HDM2 in AGS CDDPR Pool 1, AGS CDDPR Pool 2, AGS CDDPR Clone 1, and AGS CDDPR Clone 2 cells, as compared with nontreated cells (Fig. 2). To validate the data indicating that inhibition of AURKA by MLN8237 leads to decreased protein expression of p‐eIF4E (S209), c‐MYC, and HDM2 in AGS CDDPR cells, we knocked down endogenous AURKA with siRNA and evaluated the expression of p‐eIF4E and downstream effectors in these cells. Indeed, the western blot data confirmed the downregulation of p‐eIF4E (S209), c‐MYC, and HDM2 proteins following the knockdown of AURKA (Fig. 3A). The qRT‐PCR data indicated that knocking down AURKA did not decrease the mRNA expression of c‐MYC, and HDM2 in AGS CDDPR cells (Fig. 3B). On the contrary, we observed some induction of mRNA expression level (Fig. 3B) despite the reduction in their protein level, which may reflect a compensatory cellular feedback mechanism in an attempt to restore the protein expression back to its base level. Taken together, these results confirm that regulation of c‐MYC and HDM2 in resistant cells is post‐transcriptional. These results are also in line with phosphorylation of eIF4E (S209), which plays an important role in protein translation, suggesting that AURKA‐mediated cap‐dependent translation is likely a dominant mechanism. Therefore, we next investigated protein translation activity. We utilized a unique dual‐*renilla*‐firefly‐luciferase pcDNA3‐rLuc‐PolioIRES‐fLuc reporter to measure cap‐dependent/cap‐independent translation (Liu *et al*., 2013), followed by treatment with MLN8327. We found that the cap‐dependent renilla activity is higher in CDDP‐resistant cells (*P *<* *0.01), as compared to parental cells, and was significantly decreased after MLN8237 treatment in AGS CDDPR cells (*P* < 0.01) (Fig. 3C). We also measured the transcriptional activity of the induced c‐MYC protein in resistant cells using the 4xEMS c‐MYC reporter, which contains 4 c‐MYC binding sites (Boone *et al*., 2011), followed by treatment with MLN8327. The data indicated that inhibition of AURKA significantly decreased c‐MYC transcription activity in AGS CDDPR cells (*P* < 0.05) (Fig. 3D).

**Figure 4:**
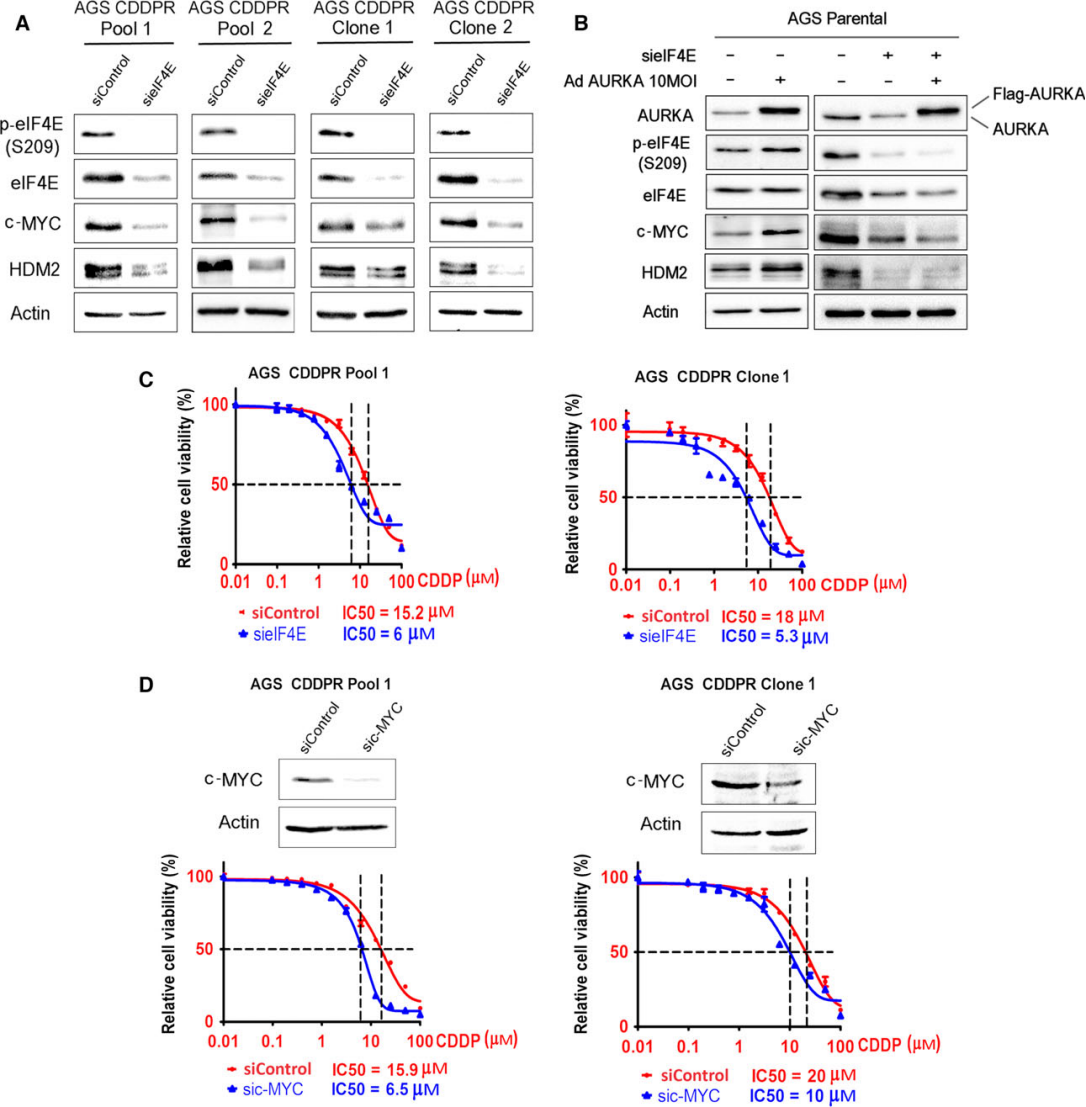
AURKA‐mediated regulation of c‐MYC and HDM2 requires eIF4E. (A) AGS CDDPR Pool 1, AGS CDDPR Pool 2, AGS CDDPR Clone 1, and AGS CDDPR Clone 2 cells were transiently transfected with control siRNA (siControl) or siRNA specific for eIF4E (sieIF4E) for 48 h. Cell lysates were subjected to western blot analysis of the indicated proteins. Knocking down of eIF4E decreased c‐MYC and HDM2 protein expression. (B) AGS Parental cells were transiently transfected by siControl + AdControl, siControl + AdAURKA, sieIF4E + AdControl, or sieIF4E + AdAURKA for 48 h. Cell lysates were then subjected to western blot analyses of the indicated proteins. The data showed that overexpression of AURKA increased protein levels of p‐eIF4E (S209), c‐MYC, and HDM2. Knockdown of eIF4E suppresses AURKA‐induced upregulation of c‐MYC and HDM2 protein expression. (C) AGS CDDPR Pool 1 and AGS CDDPR Clone 1 cells were transfected with siControl or sieIF4E for 48 h and subjected to CellTiter‐Glo viability assay. Knocking down of eIF4E significantly sensitized cells to CDDP (*P *<* *0.05), as indicated by two‐ to threefold decrease in CDDP IC50. (D) AGS CDDPR Pool 1 and AGS CDDPR Clone 1 cells were transfected with siControl or sic‐MYC for 48 h and subjected to CellTiter‐Glo viability assay. Knocking down of c‐MYC significantly sensitized cells to CDDP (*P *<* *0.05), as indicated by twofold decrease in CDDP IC50.

To test our hypothesis that AURKA‐mediated acquired CDDP resistance is dependent on eIF4E and downstream effectors, we knocked down eIF4E or c‐MYC in AGS CDDPR cells and assessed cell viability in response to CDDP. Our western blot data showed, as expected, that knockdown of eIF4E downregulated the downstream effectors, c‐MYC and HDM2 (Fig. 4A). To confirm that the regulation of downstream effectors, c‐MYC and HDM2, by AURKA is mediated by eIF4E, we transiently expressed AURKA with and without knocking down eIF4E in AGS cells. Western blot data showed that AURKA‐induced c‐MYC and HDM2 protein expression was suppressed following eIF4E knockdown (Fig. 4B), confirming that AURKA regulates these proteins in an eIF4E‐dependent manner. Notably, we observed that knockdown of eIF4E significantly sensitized AGS CDDPR Pool 1 (*P *<* *0.05) and AGS CDDPR Clone 1 (*P *<* *0.01) cells to CDDP as indicated by a decrease in IC50 from 15.2 to 6 μm and from 18 to 5.3 μm, respectively (Fig. 4C). In addition, our data indicated that knockdown of the downstream effector, c‐MYC, significantly increased the sensitivity of AGS CDDPR Pool 1 (*P *<* *0.01) and AGS CDDPR Clone 1 (*P *<* *0.05) cells to CDDP as indicated by a decrease in IC50 from 15.9 to 6.5 μm and from 20 to 10 μm, respectively (Fig. 4D). Taken together, these results indicate that AURKA‐mediated acquired CDDP resistance is dependent on eIF4E and its downstream targets in AGS gastric cancer cells and suggest that targeting AURKA can be an effective therapeutic approach in CDDP‐resistant cells.

### 3.4 AURKA enhances *de novo* CDDP resistance through regulation of eIF4E, c‐MYC, and HDM2

**Figure 5:**
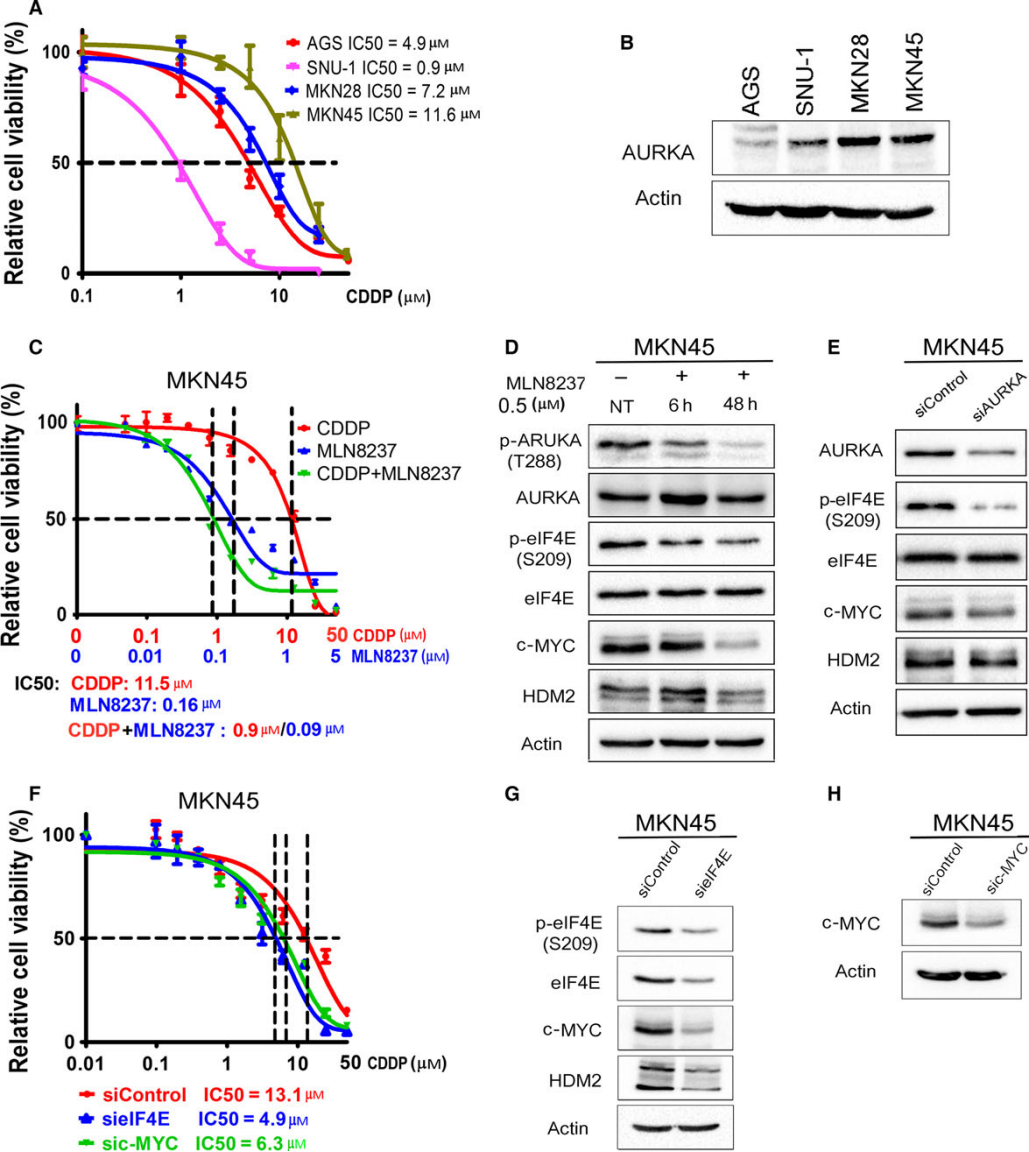
AURKA mediates *de novo* CDDP resistance through regulation of eIF4E and its downstream effectors. (A) Gastric cancer cells (AGS, SNU‐1, MKN28, and MKN45) in 96‐well plates were treated with CDDP following an 8‐point twofold serial dilution, for 5 days. Cells were then subjected to CellTiter‐Glo assay to determine cell viability. IC50 values were determined as described in Materials and methods. (B) Protein extracts from the gastric cancer cell lines, shown in panel A, were subjected to western blot analysis of the indicated proteins. The results indicated high protein levels of AURKA in cell lines with *de novo* CDDP resistance (MKN28 and MKN45). (C) CDDP‐resistant MKN45 cells in 96‐well plates were treated with CDDP, MLN8237, or CDDP + MLN8237 at a fixed ratio (10 : 1) following a 12‐point twofold serial dilution of CDDP, for 5 days. The CellTiter‐Glo assay results showed that treatment with MLN8237 alone or in combination with CDDP significantly reduced cell viability in comparison with the treatment with CDDP alone (*P* < 0.001). (D) MKN45 cells were treated with MLN8237 (0.5 μm) for 6 h or 48 h. (E) MKN45 cells were transiently transfected with siControl or siAURKA for 48 h. Cell lysates were subjected to western blot analysis of the indicated proteins. The results indicated that pharmacologic inhibition (D) or knockdown (E) of AURKA reduces protein levels of p‐eIF4E (S209), c‐MYC, and HDM2 in CDDP‐resistant MKN45 cells. (F) MKN45 cells were transfected with siControl, sieIF4E, or sic‐MYC for 48 h and subjected to CellTiter‐Glo viability assay. Knocking down of eIF4E or c‐MYC significantly sensitized cells to CDDP (*P *<* *0.05), as indicated by twofold decrease in CDDP IC50. (G,H) Cell lysates were subjected to western blot analysis of p‐eIF4E (S209), eIF4E, c‐MYC, and HDM2 proteins. Gel loading was normalized for equal β‐actin.

We next investigated whether AURKA–eIF4E axis is also present in *de novo* CDDP resistance. We first screened a panel of gastric cancer cell lines for their sensitivity to CDDP and correlation with protein expression of AURKA, p‐eIF4E, eIF4E, c‐MYC, and HDM2. Our cell viability data in response to CDDP indicated various levels of sensitivity (IC50) of the following cell lines: AGS (4.9 μm), SNU‐1 (0.9 μm), MKN28 (7.2 μm), and MKN45 (11.6 μm) (Fig. 5A). Western blot data demonstrated high levels of AURKA in CDDP‐resistant cells (MKN28 and MKN45 cell lines) (Fig. 5B). We next selected MKN45 cells, which exhibit the highest degree of CDDP resistance, relative to other cell lines, as a model of intrinsic *de novo* resistance to investigate whether targeting AURKA can achieve a therapeutic response. Cell viability data showed that MLN8237 alone or in combination with CDDP can significantly reduce cell viability as compared to CDDP alone (*P *<* *0.01, Fig. 5C). Accordingly, western blot data showed that inhibiting AURKA using MLN8237 (Fig. 5D) or AURKA knockdown using siRNA (Fig. 5E) downregulated protein expression of p‐eIF4E (S209), c‐MYC, and HDM2 in MKN45 cells, similar to acquired resistance cell models. To investigate whether AURKA‐mediated *de novo* CDDP resistance is dependent on eIF4E and c‐MYC, we knocked down eIF4E or c‐MYC in MKN45 cells and assessed cell viability in response to CDDP. Our data showed that knocking down either eIF4E or c‐MYC significantly sensitized cells to CDDP (*P *<* *0.05), as indicated by approximately a twofold decrease in IC50 (Fig. 5F). Western blot data indicated that knocking down of eIF4E markedly decreased c‐MYC and HDM2 protein levels (Fig. 5G). The knockdown of c‐MYC in MKN45 cells was confirmed (Fig. 5H). Collectively, our data demonstrate that AURKA–eIF4E axis promotes *de novo* CDDP resistance in MKN45 cells.

### 3.5 MLN8237 alone or in combination with CDDP suppresses growth of resistant cell‐derived xenografts *in vivo*

**Figure 6:**
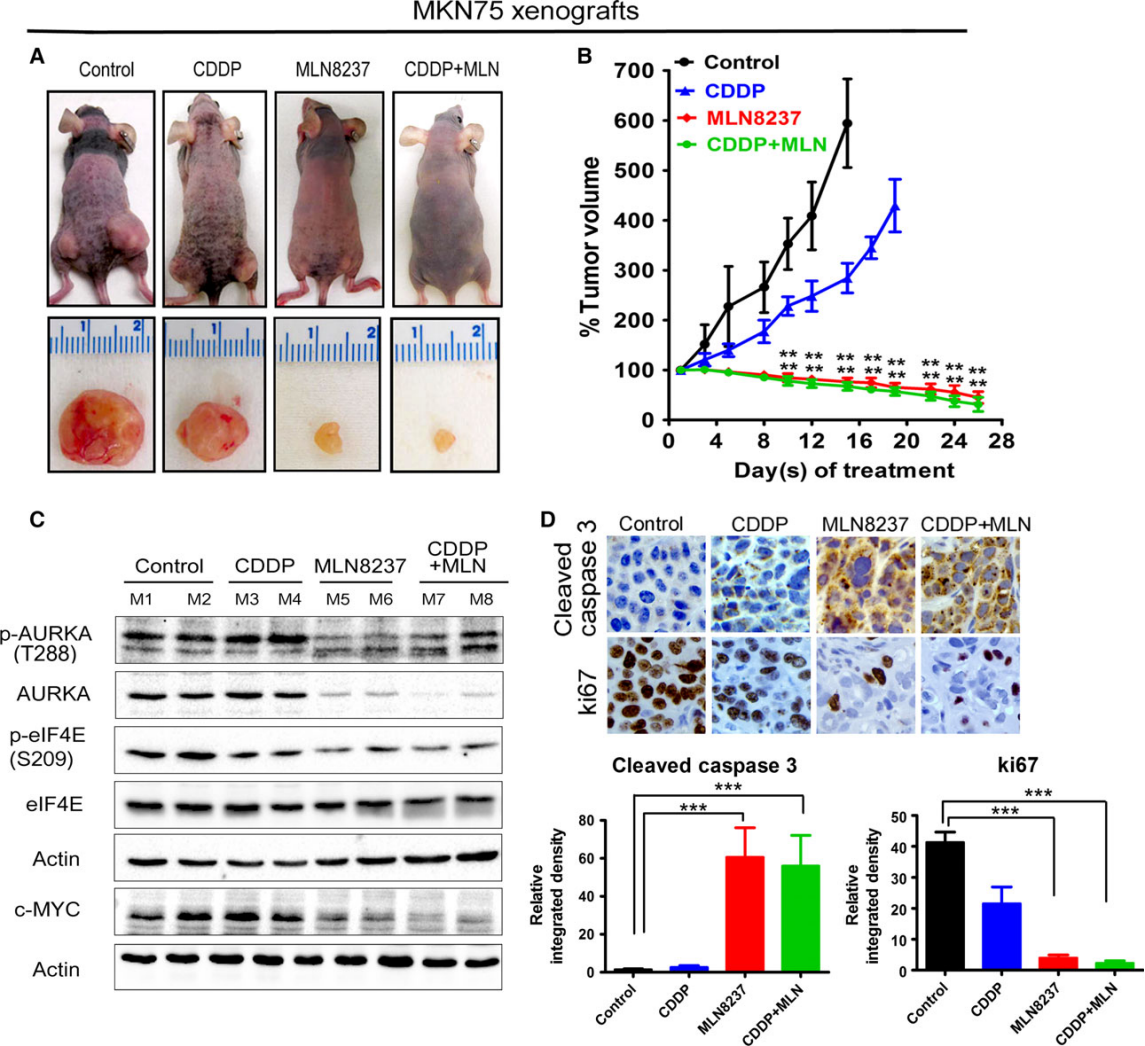
MLN8237 alone or in combination with CDDP effectively reduces resistant cell‐derived xenograft growth *in vivo*. MKN45 cell‐derived xenograft tumors (150–200 mm^3^ in size) were treated with CDDP (2.5 mg·kg^−1^, once a week) alone, MLN8237 (40 mg·kg^−1^, five times a week) alone, or with combination of CDDP and MLN8237 for 28 days, and tumor sizes were measured twice a week. (A,B) The data indicated that MLN8237 alone or in combination with CDDP has a significant antitumor activity against resistant MKN45 cell‐derived xenografts. (C) Tumor tissue lysates were subjected to western blot analysis of the indicated proteins. (D) IHC analysis of MKN45 cell‐derived xenografts showing Ki‐67 and cleaved caspase‐3 expressions *in vivo*. Relative integrated density indicates the quantification data of immunostaining intensities, which was calculated using image j software; **P < 0.01, ***P < 0.001.

We next sought to assess the *in vivo* efficacy of MLN8237 alone or in combination with CDDP using subcutaneous xenograft tumor models. The treatments were initiated after the tumor xenografts reached 150–200 mm^3^ in size, with at least 10 tumor xenografts per group. We treated the CDDP‐resistant MKN45 cell‐derived xenografts with CDDP alone, MLN8237 alone, or in combination with CDDP, and examined the tumor growth rate and protein expression levels of eIF4E, p‐eIF4E (S209), and c‐MYC in xenografts. The data showed that CDDP treatment had a relatively limited negative effect on tumor growth; however, MLN8237 significantly reduced the rate of tumor growth following 4 weeks of treatments (*P* < 0.01, Fig. 6A,B). The addition of CDDP to MLN8237 did not enhance the antitumor efficacy of MLN8237 in CDDP‐resistant cells. Western blot data analyses indicated that treatments with MLN8237 substantially decreased p‐AURKA (T288), AURKA, p‐eIF4E (S209), and c‐MYC protein levels in xenografts (Fig. 6C). Consistent with the decreased rate of tumor growth in response to treatment with MLN8237, the immunohistochemical data analysis showed a significant decrease in Ki‐67 (a marker of cell proliferation) and an increase in cleaved caspase‐3 (a marker of apoptosis) in xenografts (*P* < 0.001, Fig. 6D). Taken together, our results strongly suggest that targeting AURKA using MLN8237 can induce tumor regression and achieve a therapeutic response in CDDP‐resistant gastric cancer.

## 4 Discussion

Gastric cancer is the third cause of cancer‐related deaths in the world. Resistance to chemotherapeutic agents is a major cause for gastric cancer recurrence and low patient survival rates (Ferlay *et al*., 2015). Platinum therapy is a first‐line chemotherapeutic agent for several cancer types, including gastric cancer. However, cancer cells eventually develop acquired resistance against cisplatin (CDDP), which adversely affects patients’ prognosis (Hainsworth *et al*., 1992; Kartalou and Essigmann, 2001). Therefore, identification of novel therapeutic strategies to target CDDP‐resistant cancer cells has significant clinical implications for improving response and clinical outcome in patients with gastric cancer. In this study, we established a link between AURKA, eIF4E, and CDDP resistance. Our data demonstrate that high levels of AURKA promote CDDP‐acquired and *de novo* resistance through eIF4E phosphorylation and upregulation of its downstream effectors, c‐MYC and HDM2 proteins. The results indicate that inhibiting AURKA can be an effective therapeutic approach to target CDDP‐resistant gastric cancer cells.

Overexpression of AURKA has been associated with poor outcome and chemotherapeutic drug resistance (Sumi *et al*., 2011). At the same time, aberrant activation of eIF4E, as a mechanism of drug resistance, has been described in several cancers (Huang *et al*., 2012; Liang *et al*., 2013). Our results indicated that AURKA inhibition or knockdown downregulated p‐eIF4E (S209), c‐MYC, and HDM2 proteins in acquired and *de novo* CDDP‐resistant cells. Overexpression and activation of c‐MYC, by means of increased transcription, translation, or protein stability, is associated with aggressive tumors and poor patient prognosis (Horiuchi *et al*., 2014; Schmidt, 2004). Of note, there are accumulating lines of evidence that show that AURKA can regulate c‐MYC in cancer. We have previously shown that AURKA can regulate transcription of c‐MYC through the activation of β‐catenin (Dar *et al*., 2009). Recent studies have shown that inhibition of AURKA can regulate phosphorylation and stability of MYC, suggesting targeting AURKA–MYC axis by AURKA inhibitors as a novel potentially effective therapeutic approach (Dauch *et al*., 2016; Richards *et al*., 2016; Silva *et al*., 2014). We investigated whether eIF4E and its downstream effector, c‐MYC, mediate the function of AURKA and CDDP resistance. Our data demonstrate that knockdown of eIF4E or c‐MYC significantly sensitized acquired or *de novo* resistant cells to CDDP. Therefore, our results add to the current knowledge and suggest a multifaceted regulation of c‐MYC at different levels by AURKA in cancer. We and others have previously shown that AURKA can also regulate the key ubiquitin ligase involved in the degradation of p53, HDM2, whereby overexpression of AURKA promoted HDM2 phosphorylation and enhanced its stability leading to the degradation of p53 (Hsueh *et al*., 2013; Sehdev *et al*., 2014; Vilgelm *et al*., 2015). Collectively, our new findings in this report add a significant novel perspective by showing that activation of AURKA–eIF4E axis is required for the induction of c‐MYC and HDM2 protein levels and CDDP resistance in gastric cancer cells. Of note, a recent study has shown that AURKA can mediate resistance to mTOR inhibition by everolimus (Katsha *et al*., 2017). This involved AURKA‐dependent activation of eIF4E through the inhibition of PP2A activity. Although we did not investigate this signaling axis in detail in CDDP resistance, it is possible that AURKA‐mediated regulation of PP2A is involved in the activation of eIF4E in CDDP resistance, too. Taken together, activation of AURKA–eIF4E axis in cancer cells could play a crucial role for resistance to several chemotherapeutics.

A previous report indicated that AURKA was implicated in CDDP resistance in murine bone marrow Ba/F3 cells transformed by JAK2 V617F mutant (Sumi *et al*., 2011). Another study indicated that AURKA suppresses CDDP‐induced apoptosis through phosphorylation of p73 and its sequestration in the cytoplasm in cancer cells (Katayama *et al*., 2012). Of note, earlier reports have shown that AURKA can suppress p73 (Dar *et al*., 2008a; Katayama *et al*., 2012) and activate STAT3, β‐catenin, and NF‐κB (Dar *et al*., 2009; Katsha *et al*., 2013, 2014). Although we have not specifically investigated these pathways in the present study, it is plausible that targeting AURKA in CDDP‐resistant cancer models may induce cancer cell death not only through the suppression of eIF4E‐c‐MYC‐HDM2, but also through reversing these signaling effects.

## 5 Conclusions

In summary, we present evidence for a novel mechanism by which AURKA promotes resistance to CDDP through the activation of p‐eIF4E, c‐MYC, and HDM2. Targeting AURKA using MLN8237 may present a clinically relevant opportunity to treat CDDP‐resistant tumors and enhance the therapeutic response in patients with gastric cancer.

## Author contributions

LW and JA performed the main experiments, summarized the results, and wrote the manuscript. AK and SH assisted in performing the experiments. AB assisted in interpretation and troubleshooting of the data. JE assisted in obtaining MLN8237 and in revising the manuscript. WER provided the concept, funding, supervision, and assisted in writing the manuscript. All authors read and approved the final manuscript.
